# From novice to master: a three-phase developmental study on the self-efficacy of traditional handicraft artisans

**DOI:** 10.3389/fpsyg.2026.1777317

**Published:** 2026-03-25

**Authors:** Ying Chen, Hui’e Liang, Lingyun Fang

**Affiliations:** 1 Jiangsu Province Intangible Cultural Heritage Research Base, Jiangnan University, Wuxi, Jiangsu Province, China; 2 Wuxi University, Wuxi, Jiangsu Province, China

**Keywords:** phases, self-efficacy, sources, stem education, traditional crafts

## Abstract

To achieve the Sustainable Development Goals (SDGs), modern STEM education urgently needs to integrate learners’ cognitive, affective, and behavioral development within complex practical teaching. However, the understanding of the underlying psychological evolution mechanisms in such long-term practice remains insufficient. Notably, these educational concepts have long been substantively embodied and practiced within the traditional handicraft apprenticeship model, through which numerous highly skilled artisans have been cultivated. Consequently, the learning journey of traditional handicraft artisans from novice to master serves as an ideal, historically rich, and demonstrably effective case for investigating the aforementioned psychological mechanisms. To uncover the core psychological dynamics underpinning the skill refinement of traditional handicraft artisans, this study focuses on the sources and development of self-efficacy. Guided by grounded theory, semi-structured retrospective interviews were conducted with 10 senior artisans, leading to the construction of a three-phase model comprising nine key themes related to the sources of self-efficacy. The findings reveal significant phase-specific differences in the sources of artisans’ self-efficacy along their skill development: (1) In the foundational mastery phase, it primarily stems from affective states, vicarious experience, and mastery experience. (2) In the proficiency and application phase, the core sources shift to external support, vicarious experience, and affective states. (3) In the breakthrough and innovation phase, the emphasis is on self-regulation, physiological and affective states, and mastery experience. Specifically, the concept of “social persuasion” proposed by Bandura manifests more as “external support” during the proficiency phase within the context of handicraft practice. Meanwhile, the “physiological state” dimension within “physiological and affective states” appears less salient in both the foundational acquisition and proficient application stages. Furthermore, “self-regulation” emerges as a distinct source, emphasizing autonomous authority over the creative process. This extends beyond skill-based confidence, constituting a key characteristic of the innovation phase. Based on these findings, this study proposes corresponding program design implications for each phase: the foundational phase should prioritize creating a guidance-oriented exploratory environment; the proficiency phase necessitates building an application platform connecting diverse resources; and the innovation phase should focus on constructing a meaning-making community with a high degree of autonomy. This research not only deepens the understanding of Bandura’s self-efficacy theory within the unique context of long-term skill acquisition by traditional handicraft artisans but also provides a psychological perspective for designing phased skill cultivation programs in this field.

## 1 Introduction

The interdisciplinary and inquiry-based practical learning model inherent in traditional handicrafts demonstrates a high degree of consistency with contemporary STEM education concepts (Al Hamad et al., 2024). Firstly, traditional handicrafts themselves encompass a rich interdisciplinary knowledge structure. Their specific practices often integrate materials science, technological tools, structural engineering, and mathematical principles. For instance, the production of handmade paper requires precise mastery of raw material ratios, involving the application of materials science (Ihwah et al., 2021). To create handicrafts that align with modern aesthetics, targeted research and improvement of tool technology are essential (Rantšo, 2022). When crafting gemstone-based artworks, understanding the material’s optical properties and internal structure is necessary, which relates to knowledge in structural engineering (Sodo et al., 2003). Similarly, the process of weaving rattan handicrafts relies on the support of mathematical knowledge (Prastika and Abidin, 2021). Secondly, the transmission of traditional handicrafts is highly dependent on practical inquiry. Currently, its inheritance presents two main types: natural social transmission and formal higher education transmission (Song et al., 2024). Natural social transmission serves as the historical core pathway for craft continuity, primarily relying on two models: kinship-based family inheritance and socially-based master-apprentice inheritance (Rivero, 2016). As traditional natural social transmission faces contemporary challenges such as fragile inheritance chains, limited dissemination efficiency, and relatively closed knowledge systems, traditional handicraft education has actively moved towards formalized learning pathways (Johnson and Majewska, 2022). Through integration into vocational institutions, establishment of specialized courses, and development of standardized teaching materials, systematic and scalable transmission paths are being explored (Man, 2018). Given that craftsmanship itself constitutes a highly personalized form of tacit knowledge, inherently implicit and non-coded, dependent on practice and experience (Van Kampen, 2019), and its evaluation is often embedded in the quality of the work, industry recognition, and the tacit understanding between master and apprentice (Agarwal et al., 2004), any educational transmission model necessitates a practice-based approach to ensure the effective transfer of such difficult-to-articulate skills and insights.

While this alignment lays a theoretical foundation for integrating traditional crafts with modern education, current research remains limited in its perspectives. On one hand, studies from a socio-anthropological perspective focus on analyzing how crafts are generated and evolve within specific social networks and cultural meanings, aiming to explain their local logic and provide references for macro-level policies (Yang et al., 2022; Karakul, 2015). On the other hand, at the level of educational practice, concrete attempts have emerged, such as designing craft curricula based on the STEAM education framework (Ji et al., 2019b) and utilizing emerging technologies to enhance teaching experience and effectiveness (Ji et al., 2020), reflecting the active integration and innovation of teaching concepts and methods during the formalization process.

In contrast, research from a cognitive psychology perspective is relatively scarce. Given the long-term nature of craft cultivation, neglecting psychological support can easily lead learners to abandon their studies due to prolonged frustration, ambiguous goals, or a loss of meaning. According to the Talent Development Megamodel (TDMM) theory, socio-psychological skills are key drivers of talent development, particularly at advanced stages, where they serve as the differentiating factor between proficiency and excellence (Olszewski-Kubilius et al., 2023). Instructors can foster the formation of such skills by providing practical opportunities and timely guidance. Among these, self-efficacy is a typical and crucial psychological variable, influencing the mastery of complex knowledge, the ability to address technical challenges, and the persistence to innovate (Bandura, 2006). Self-efficacy refers to individuals’ judgments of their capabilities to organize and execute the courses of action required to attain designated types of performances (Bandura, 2006). This theory has been widely applied in educational fields, either as a direct research subject or as a key mediator in professional learning processes, leading to diverse talent cultivation practices across disciplines (Zimmerman, 1995).

Although self-efficacy theory has been validated in multiple professional education contexts, its mechanisms and cultivation pathways within the specific context of traditional craft inheritance remain underexplored. The transmission mode of traditional crafts relies heavily on individual experience and belief systems, offering a unique and significant context for studying self-efficacy. Therefore, from a cognitive psychology perspective, this study aims to (1) analyze the self-efficacy of master artisans in traditional craft fields to uncover the belief formation mechanisms underlying their expertise, and (2) based on this theoretical foundation and integrating TEDM educational principles, propose concrete project design strategies for cultivating novice artisans, thereby promoting innovation in craft education practice.

## 2 Literature review

### 2.1 Sources of self-efficacy in traditional handicraft artisans

Self-efficacy refers to an individual’s belief in their own capabilities and serves as a core force guiding behavior, sustaining persistence, and enabling achievement. It plays an indispensable role throughout the extended developmental journey of traditional artisans. On the one hand, artisans are more inclined to engage in tasks they believe they can accomplish and less likely to undertake those they perceive as beyond their competence. On the other hand, this affirmation of their own abilities enables them to return to their tools despite repeated setbacks and empowers them to venture beyond established conventions to attempt subtle innovations. Without such internal conviction, it is often difficult to maintain the focus and resilience required to endure prolonged and tedious practice, confront the challenges of complex techniques, and navigate market uncertainties. Thus, self-efficacy not only influences their specific behavioral choices but also constitutes a crucial psychological foundation that underpins the entire process of skill acquisition and refinement.

Therefore, understanding and shaping self-efficacy is crucial for promoting the learning and development of traditional handicraft artisans. It has been suggested that teachers aiming to enhance students’ academic and self-regulatory self-efficacy should first focus on the sources underlying these beliefs (Woolfolk and Shaughnessy, 2004). According to Bandura (1986, 1997), self-efficacy stems primarily from four sources: mastery experience, vicarious experience, social persuasion, and physiological and affective states. Mastery experience, one of the most influential sources, relates directly to an individual’s past successes. Previous achievements can significantly strengthen one’s confidence in their capabilities. Vicarious experience involves learning and adjusting self-assessments by observing the successes or failures of others. Social persuasion refers to the process whereby individuals’ willingness to invest effort and persist in the face of difficulties is enhanced through verbal or non-verbal positive social feedback and evaluations from others, thereby fostering the continued development of both competence and self-efficacy. Physiological and affective states function as a source of self-efficacy, whereby individuals judge their capability to successfully manage specific tasks or challenges by evaluating and interpreting their own bodily reactions, such as tension or fatigue, and emotional experiences, such as excitement or discouragement; positive states typically contribute to higher self-efficacy.

Self-efficacy beliefs are most likely to change during skill development, particularly when traditional handicraft artisans encounter novel tasks. Research has observed that these changes in self-efficacy interact with its sources over time (Peura et al., 2021). Furthermore, phased exploration has been conducted regarding the formation of self-efficacy among teachers in the early stages of their careers (Gale et al., 2021). Studies have also confirmed that the relative influence of different self-efficacy sources varies in priority when applied to distinct populations (Zeldin and Pajares, 2000). Consequently, understanding the sources of traditional handicraft artisans’ self-efficacy involves two key aspects: first, these sources evolve over time along with the individual’s developmental journey; second, at any given point, the influence of different sources varies in strength. Of note, underlying this dynamic variation and differential salience is the individual’s process of cognitive appraisal regarding information sources. Information can only exert an authentic influence on efficacy beliefs through individuals’ cognitive appraisal and meaning-making. In other words, the same objective event may produce vastly different efficacy effects for different individuals, or even for the same individual in different contexts, due to differences in how it is noticed, interpreted, weighted, and retrospectively reconstrued. It is precisely this appraisal process that shapes the fluctuating salience of various sources and constitutes the micro-level psychological mechanism underlying the dynamic development of self-efficacy.

In summary, as a key psychological mechanism driving the development of traditional handicraft artisans, the formation of self-efficacy is dynamically evolving. Therefore, this study will examine in stages which sources of self-efficacy play a predominant role during different periods of the artisans’ skill development.

### 2.2 The three-phase development of traditional handicraft artisans

Education and training programs are widely recognized as a vital pathway for transmitting traditional skills from accomplished practitioners to new generations, as evidenced by practices in many countries. For example, Lithuania has consistently incorporated traditional handicraft instruction as a key component of its national curriculum over recent decades (Sederevičiūtė-Pačiauskienė et al., 2019). Similarly, students in Finnish comprehensive schools continue to concentrate on developing technical making skills (Autio, 2016). Given the multifaceted value of traditional handicrafts, a holistic approach to cultivation is essential. From an environmental perspective, handicrafts often demonstrate sustainable characteristics compared to mass-produced goods, as they typically utilize natural and recyclable materials, require lower energy input, and involve less polluting production processes (Reddy and Raipally, 2014). Socially, they provide significant employment opportunities, particularly in rural areas, serving as an effective means to mitigate unemployment and poverty (Din, 2017; Islam et al., 2020). In cultural and economic terms, handicrafts can reinforce ethnic cultural identity and enhance women’s socioeconomic status and participation through craft production (Schilar and Keskitalo, 2018; Bano et al., 2021).

Therefore, cultivating new generations of practitioners necessitates a dual-level strategy. At the popularization level, aimed at the general public, efforts should focus on cultural dissemination and value formation, enabling groups such as university students and visitors to meaningfully engage with the craft’s core principles. This can be facilitated through informal education that encourages civic participation in local cultural governance (Yan and Chiou, 2021). At the professional level, a systematic training system must be established for aspiring artisans, emphasizing deep skill transmission and sustainable career development. Scholars such as Karakul (2015) have proposed integrated methodologies for conserving traditional craftsmanship, including the documentation of both physical structures and associated cultural expressions. While these two levels differ in their target audiences and objectives, they jointly constitute the foundation for the sustainable development of talent in the field of traditional handicrafts.

Regarding curriculum design within learning programs, Pöllänen (2009) argues that school-based craft education should move beyond traditional models by implementing a holistic craft concept, thereby situating teaching within more authentic environments and activities. Dang et al. (2021) further propose collaborating with local enterprises. The research team led by Ji has long been dedicated to utilizing augmented reality (AR) to achieve personalized learning modes in traditional handicraft education (Liu et al., 2020). In 2019, the same team advanced the use of WebAR technology to address the limitations of existing AR applications in this learning context (Ji et al., 2019a). Additionally, building upon five key elements of interactive behavior, they constructed a new model for interactive learning experiences in traditional handicrafts (Ji et al., 2018). Concurrently, Zhu et al. (2024) integrated digital games with craft practice to create an embodied experiential educational game model, aiming to balance engagement and enjoyment with effective heritage learning. These studies, focusing on the learners themselves and encompassing aspects such as learning forms, pathways, and mechanisms, aim to analyze the external frameworks and operational processes that enable the transmission of craft skills.

In recent years, a subset of research has gradually turned towards the intrinsic cognitive dimensions within the transmission process. For instance, integrating perspectives from linguistic theory and neuroscience, Marchand (2008) revealed that learning a craft is not merely dependent on external training but constitutes an internal process of cognitive deepening. Through sustained bodily practice, learners achieve a cognitive transition from unfamiliarity to familiarity and from clumsiness to mastery. In Sweden, for example, establishing a traditional handicraft shop requires first completing an apprenticeship, then working for 3 years as a journeyman, and finally having one’s exemplary work recognized by the guild (Edgren, 1986). This progression can be broadly delineated into three key phases. The initial apprentice phase involves mastering the essentials of the craft through imitation and foundational training. The subsequent journeyman phase, characterized by independent operation, entails a comprehensive understanding of the entire workflow, where stable skill is formed and understanding deepened through continuous bodily practice. The culminating master phase represents a stage of integration, where individuals not only possess consummate skill but also the capacity for innovation and transmission. It must be emphasized that these phases are not separated by clear temporal boundaries but represent an interwoven and gradual transitional process. This delineation aims to clarify that different core competencies are required at different stages, which inevitably leads to variations in individuals’ beliefs in their own capabilities. These evolve from a belief in one’s ability to learn during the foundational phase, to a belief in one’s ability to perform well during the proficiency phase, and finally to a belief in one’s ability to create during the breakthrough and innovation phase.

### 2.3 STEM education and self-efficacy

The research by Vieira et al. (2025) identifies creative self-efficacy as a significant component of STEM education. This finding underscores the importance of students’ intrinsic psychological cognition within STEM learning. Furthermore, Kiran and Sungur (2012) note that understanding patterns of self-efficacy within STEM fields constitutes the foundational step toward developing solutions for challenges in this domain. It is important to emphasize that an individual’s self-efficacy is highly dependent on specific learning contexts, and analysis from a generalized perspective should be avoided (Kaufman, 2006). Therefore, the essential first step is to achieve a deep, contextualized understanding of the mechanisms of self-efficacy within STEM education. Subsequently, targeted and effective cultivation strategies can be designed. This approach is vital for effectively addressing practical challenges in STEM education, such as student aversion to difficulties and insufficient innovative capacity, thereby genuinely enhancing its quality and outcomes.

A highly insightful perspective emerges from the instructional model of traditional handicrafts. Analysis reveals that although this model is not explicitly labeled “STEM,” it inherently embodies its core educational philosophy. Specifically, through the continuous cycle of designing, crafting, debugging, and refining authentic, cross-contextual artifacts, it comprehensively cultivates learners’ knowledge, skills, and thinking. The protracted journey of traditional master artisans—progressing from observation and imitation to independently completing works of excellence and innovation—is precisely the process through which they gradually build and solidify their self-efficacy. Consequently, studying this process can provide us with a time-tested psychological blueprint for how to consciously design contexts to foster self-efficacy within modern STEM education.

In summary, the progression of traditional handicraft artisans from apprentices to masters across three phases constitutes not merely a process of technical refinement, but also a dynamic process of psychological construction. Within this journey, the sources of self-efficacy and their influences evolve alongside the developmental phases. Consequently, investigating the specific mechanisms through which these sources operate at different phases is crucial for understanding the formation and sustenance of artisan confidence. With the advancement of educational psychology and related learning sciences research, there is a clear need to design effective phased cultivation strategies based on the aforementioned psychological mechanisms. These strategies aim to enhance the self-efficacy of artisans, thereby facilitating their development into outstanding talents capable of autonomous innovation.

To achieve this objective, the following three specific research questions are proposed:

1. What are the primary sources of self-efficacy for traditional craft learners at different stages of their development?
2. How does strong self-efficacy concretely influence the progression of a learner’s technical skills?
3. Based on the identified sources and influencing mechanisms, what program strategies should be formulated within the STEM educational framework to enhance the self-efficacy of handicraft learners?

## 3 Materials and methods

### 3.1 Methods and procedures

This study employed qualitative methods to investigate the sources of self-efficacy. Qualitative research, exemplified by semi-structured interviews, effectively captures the development of self-perception by allowing participants to elaborate on experiences most significant to them over time (Usher and Pajares, 2008). Personal accomplishment is the most reliable source of efficacy information. By conducting semi-structured interviews with eight experienced educators, Bi et al. collected concrete evidence of their successes, thereby providing targeted strategies for developing students’ self-efficacy skills (Bi and Rauduvaitė, 2025). Moreover, the growth of senior artisans often spans decades, and the refinement of their skills is intricately interwoven with personal life development and historical change, making it irreducible to a few metrics. Furthermore, the essence of their core craftsmanship is often deeply embedded in bodily memory and affective experience. In summary, to comprehend such a complex and profound developmental journey, researchers must, with considerable patience, guide artisans through profound recollection and detailed narration.

Based on the stage-specific characteristics exhibited by traditional handicraft learners, this study focuses on the entire growth process and constructs an analytical framework for the development of self-efficacy. The research intends to conduct in-depth interviews with outstanding master artisans, using a retrospective approach to explore the sources and evolution of their self-efficacy. The grounded theory method will be applied to code and analyze the interview data. Finally, based on the findings, suggestions will be proposed for project design in the cultivation of traditional handicraft talents. Grounded theory offers distinct advantages in exploratory research, particularly during the early phase of investigating the sources of self-efficacy in the traditional handicraft field (Glaser and Strauss, 2017). The data analysis in this study primarily involves two core stages: open coding and axial coding, aimed at refining categories and their interrelationships. Following this, a saturation test is required before a new theoretical framework can be finalized (Henninger et al., 2017).

### 3.2 Data collection

A recognized master artisan must demonstrate excellence and exemplarity in four key aspects. First, they must possess profound skill and a distinctive personal style. Second, they should have created representative classic works. Third, they need to exhibit an innovative spirit that builds upon tradition to advance the craft. Fourth, they must be committed to cultivating successors and enjoy high prestige and influence both within and beyond the industry. These attributes align with the criteria for the designation of Representative Inheritors of Intangible Cultural Heritage in China. In 1993, UNESCO recognized masters possessing a high degree of knowledge and skill as bearers of living cultural traditions (Karakul, 2012). The organization further encourages and guides countries to establish their own national systems. In response to this global framework, China established the system for Representative Inheritors of National Intangible Cultural Heritage Representative Items. As of March 2025, there were 713 national-level representative inheritors in the traditional skills category, supplemented by a substantial number of provincial and municipal inheritors forming a transmission echelon. This study considers these designated inheritors as ideal subjects for exploring talent development pathways. Their growth trajectories and competency structures provide a clear empirical foundation for understanding how to systematically cultivate such high-caliber artisanal talent.

Given the inherently small population of senior traditional master artisans, they constitute a hard-to-reach sample group for research. Therefore, all participants in this study were directly contacted and invited through personal networks. Interviews were conducted between June and December 2025, utilizing a combination of telephone and face-to-face formats. Existing research indicates that telephone interviews can yield data quality comparable to face-to-face interviews, with no significant difference in data collection effectiveness between the two methods. Furthermore, telephone interviews offer greater scheduling flexibility and may reduce potential pressure on interviewees associated with in-person interaction (Holt, 2010). Based on these considerations, this study flexibly employed telephone interviews where circumstances permitted.

The domain of traditional handicrafts encompasses a vast and complex system, consisting of 15 major categories, 106 sub-categories, and 765 specific types, with practitioners widely distributed (Hua, 2021). To address this diversity, this study purposively selected representative master artisans from distinct fields, namely textiles and carving/printing, as research participants. Integrating practical experiences from these varied domains provides substantial support for constructing a theoretical model with broader applicability. To enhance question clarity and effectively manage the interview process, a pilot interview was conducted with one participant prior to formal data collection. Insights from this pilot were used to refine the wording and structure of the interview protocol (Sutton and Austin, 2015). During the textual analysis of the interview data, no new themes emerged from the content provided by the final two participants, indicating that theoretical saturation was reached and the data collection phase could be concluded. The final participant group comprised 10 senior master artisans from fields including textiles and carving. Detailed demographic information is presented in Table 1. Each formal interview lasted approximately 120 to 150 min. This duration was designed to ensure sufficient depth of information while also considering the efficiency of the interview process and the participants’ time and energy.

**Table 1:** Demographic information of respondents.

Categorical identities	Basic information	Quantity	Percentage
Gender	Female	5	50%
	Male	5	50%
Grade of Representative Heritage Holder of Intangible Cultural Heritage of China	National level	2	20%
	Provincial level	5	50%
	Municipal level	3	30%
Craft Categories	Weaving, Dyeing, Embroidery and Clothing Making	6	60%
	Carving and Printing	4	40%

This study developed an interview guide comprising 12 core questions, structured around the three developmental phases of artisan growth. The details are presented in Table 2. Firstly, prior to the interviews, the publicly available background information of each participant was systematically reviewed. This preparatory work facilitated the researcher in conducting concrete and detailed follow-up inquiries during the interviews. The interview content involved retrospective accounts of experiences spanning several decades. To assist participants in structured and in-depth recollection, they were explicitly informed beforehand that the questions were organized into three parts. These corresponded to Phase 1 (Mastering Fundamentals), Phase 2 (Proficiency and Application), and Phase 3 (Breakthrough and Innovation). The defining characteristics of each phase were clearly explained. Questions 1, 5, and 9 served as entry points, initiating the conversation by asking participants to recall specific, vivid, and concrete scenarios. This approach aimed to facilitate ease into the dialogue, establish rapport, and lower psychological defensiveness. Building on this foundation, Questions 2, 7, and 11 were designed to focus the discussion further by guiding participants to identify the salient difficulties they encountered within the described situations, thereby enhancing the relevance and specificity of the conversation. Questions 3, 4, 6, 8, 10, and 12 were then deployed to delve into the difficulties mentioned, prompting participants to retrospectively explore their psychological state and specific actions when facing those challenges. This included probing what led them to perceive the difficulty as surmountable and what concrete coping measures they actually employed. Throughout the interview, the sequence of questions was flexibly adapted to align with the participant’s narrative flow. The overarching objective of this structured yet flexible progression was to guide the dialogue naturally from the description of external events to the exploration of internal cognitive processes. This method facilitated detailed recollection of the psychological shifts occurring during problem-solving, enabling a transition from surface-level description to in-depth narrative. Furthermore, all questions were phrased in everyday language, deliberately avoiding professional jargon. This was done to minimize interference with participants’ natural expression, thereby ensuring the authenticity and integrity of the collected data.

**Table 2:** Interview questions.

Phase	Question
Mastering fundamentals	1. What experiences prompted your decision to pursue this craft?
	2. What did you find most challenging when initially learning this skill?
	3. What motivated you to persist, rather than give up, when facing this challenge?
	4. What specific actions did you take at that time to overcome these difficulties?
Proficiency and application	5. After transitioning from apprentice to an independent craftsperson, what new and higher demands did you face?
	6. How did you go about learning and improving yourself to meet these higher demands?
	7. What was the most formidable obstacle you encountered during this phase of advancement?
	8. What led you to believe it was possible to overcome this challenge?
Breakthrough and innovation	9. In your view, what is the most important capability for becoming a senior master artisan?
	10. What targeted efforts or training have you undertaken to cultivate this key capability?
	11. In the process of achieving breakthroughs and innovation, which aspect did you consider most difficult?
	12. In your view, what factors were primarily associated with the eventual resolution of this confusion?

### 3.3 Data analysis

#### 3.3.1 Open coding of data across phases

With the assistance of Nvivo15, all original interview transcripts underwent line-by-line analysis. Statements selected for coding were identified based on the following two criteria: (1) Regarding the specific measures taken by traditional craftsmanship masters to overcome difficulties and their inner cognitive perspectives. This means that the data presented are not merely objective records of historical events, but rather the participants’ current understanding and narrative reconstruction of these events. In the process of analysis, we did not attempt to forcibly separate the “objective occurrence” of the sources from the participants’ “subjective appraisals.” Instead, we regarded this narrative, which encompasses the individuals’ cognitive appraisals, as the core object of our study. This is because we believe that it is precisely this “perceived source,” imbued with meaning by the individual, that directly constitutes the psychological reality of self-efficacy development. (2) the same core viewpoint or experience must be mentioned at least twice within the same participant’s narrative. This phenomenon emerged consistently throughout the in-depth interviews with the traditional artisans. The design of Questions 1, 5, and 9 guided participants into extended and detailed recollective narratives, while subsequent questions prompted them to re-elaborate and expand upon key situations within that narrative context. These repeated articulations precisely indicate the cognitive significance of the experience for the participant, thus justifying their extraction as valid units of analysis. Based on the above criteria, a total of 231 raw sentence labels were obtained across the three phases. After merging and conceptualizing these labels, 60 concepts were extracted. From these concepts, 26 subcategories were further summarized, with the results presented in Table 3.

**Table 3:** Open coding results for the three phases.

Phase	Subcategory	Concept	Example of raw statement
Mastering fundamentals	Familial and national duty	Organizational assignment, goal commitment, earning money, providing care	There was an urgent need for apprentices, so I was assigned by the institute. I did not think much about plans; I just knew I should follow the organization’s arrangement and learn it well. That is why I never considered giving up when facing difficulties (C1). To secure this foreign trade order, I thought I must produce it at that time (C3). It was not easy for my parents to secure this job for me; I must cherish this opportunity no matter what. How could I possibly quit halfway? (C8). I could take care of my family while doing this work. That was the reason I chose this path, otherwise, I would not have taken it on (C5).
	Past experience	Familiarity, interconnectedness	This sense of familiarity with silk threads gave me more confidence in learning this craft well (C1). Once I got the hang of it a little, I felt a sense of accomplishment, and my confidence grew (C3).
	Step-by-Step demonstration	Decomposing techniques, imitative operation, complete demonstration	Once I could control the fabric width and the woven piece looked neat, I tried simple patterns. How could I progress without taking it step by step? (C2). At that time, I wasn’t very good with a brush either; I just traced the pattern, and after tracing, I wove according to it, which made the whole process seem less difficult (C1).
	Sustained practice	Repetition, patience	If not thousands of times, I practiced at least hundreds, and with each attempt I improved, which made me more confident (C9). I spent several months just on basic skills. I was quite patient (C10). To make a simple circle or square, I had to explore for several days. It wasn’t a job for someone impatient (C2).
	Environmental immersion	Family, neighbors	Because our family originally wove homespun cloth, which shared similarities with this kesi technique, so I wasn’t too worried about being unable to learn it (C3). After school, I had nothing to do, I would go to his (neighbor’s) house. Later on, when I ran into problems, I had actually seen or tried them before, so I already had some idea how to handle them and wasn’t too worried (C7).
	Guidance from others	Instruction, suggestion	Teacher Jiang at school, he instructed me, or I would not have continued with this (C7). A photographer came to my home to photograph my work and gave me suggestions, That alone completely solved the problem. If it had not been for his suggestion, I think I would have gradually given up back then (C9).
	Driven by interest	Interest, liking, passion	I was genuinely very interested, and whenever I encountered a problem, I enjoyed figuring it out on my own (C7). I had this fondness in my heart, so it never felt difficult. In fact, I truly enjoyed it (C8).
	Educational discipline	Family uphringing	My mother cultivated my habit of self-reliance in exactly this way, so whenever I face difficulties, I never once think of retreating, and that applies to everything. (C3).
Proficiency and application	Imitation and comparison	Benchmark learning, mutual comparison	Watching him work, that level of precision, that sense of thoroughness, felt different again. We would place two similar finished pieces together for comparison, and the shortcomings became immediately clear. During that time, I often went to look at his work and learned a great deal (C1). The other party would give you a reference, and then we would produce it based on our experience. Having a reference made it manageable, unlike some clients whose requests came purely from their imagination. They were so vague you could not even grasp what they meant. (C8).
	Recognition through competition	Participating in competitions, exhibiting works, bidding	Experts were organized to evaluate and see which unit could meet their standards. To meet that benchmark, we always gave our all and persevered no matter how difficult it was (C8). Exhibitions followed one after another, and my works were published, so my mood was different then; I became more and more motivated, and there was no time to be afraid (C7).
	Personal standards	Perfectionism, steadiness and peace of mind	The master thought it was generally acceptable, but I always felt it could be done better and insisted on taking it apart to re-weave. This ‘meticulous’ drive is more about human nature, a matter of character. Seeing imperfections, I must correct them (C1). Doing my own work solidly and reliably (C10). More importantly, it’s my own internal standard (C2).
	Material support	Rewards and remuneration, family support	This rating was linked to bonuses, and everyone cared about it (C1). There always needs to be support from the family (C9). All my paper-cuttings sold out, and my interest surged again; I thought I must continue (C7).
	Encouragement and recognition	Praise, recommendation, purchase, acknowledgment	I initially thought the master would find me annoying, but unexpectedly, they were approving and thought I was eager to learn (C1).
	External supervision	Inspection, requirements	To ensure quality, the factory developed its own set of inspection standards. When products came off the loom, the inspection department would check them, grading them as first, second, or third class. This was something everyone took seriously, myself included (C1). He (the master) had high requirements for every process, so that over time, I grew accustomed to it. (C8).
	Seeking help from others	In-Person consultation, online inquiry, proactive initiative	I have one strength: At every step, I consulted these people (C2). Later, after persistent requests, the factory subsidized me and allowed me to go as well (C1). Watching their videos online, and it was this very experience that led my leaders and colleagues to take my ideas more seriously, giving me more say in matters. (C9).
	Government emphasis	Organizing events, formulating policies	The provincial department notified the cultural center for us to participate, and we all responded with exceptional enthusiasm (C7). After the concept of intangible cultural heritage emerged, I just wanted to do it well and meticulously (C9). The university organized a training program for intangible cultural heritage inheritors, which was really helpful for me back then (C1).
Breakthrough and innovation	Broad learning and understanding	Conceptualizing and searching, understanding and thinking	When actually weaving, one still has to rely on one’s own understanding and improvisation (C1).
	Obsession and addiction	Entering dreams, immersion	Constant thinking, even while sleeping, so heaven sends you dreams (C7).
	Desire for recognition	Acknowledgment	I always hope to create works that everyone can acknowledge, not content to stay at the original level (C1). If I have 10 pieces that are unanimously recognized as good, that’s enough for me (C7).
	Technological empowerment	Computer assistance, adding chemical agents	I tried using a computer to draw for the first time, and many of my larger-scale works that followed were created this way. They would have been difficult to complete otherwise (C7). Later, preservatives were added; otherwise, it would easily spoil. There would be little point in re-creating it (C9).
	Resource investment	Self-funded creation, abundant time	I was young then, with good eyesight, which allowed me to complete such work (C1). Now that there are fewer restrictions, I create according to my own aesthetic judgment and am willing to invest however much time is required. When I was still a trainee artisan, all choices were bound by my master’s directives. The materials, the time, even the design—every aspect was specified (6).
	Physiological states	Visual acuity, manual steadiness	Back when I was young and my eyes were still sharp, I was capable of creating work like this (1). Now, my hands cannot even hold a blade steady. One carve and it breaks. So when people call me a ‘master,’ my heart is heavy with guilt. It’s nothing like when I was working on that piece. My hands were steady and strong, and the carving was just right (voice breaking) (7).
	Careful observation	Seeing clearly, observing	At this stage, it’s not about learning something, but about our observational skill (C9).
	Courage and belief	Persistence, sentiment, reluctance to abandon	Reverence for culture, trust in society, and a reluctance to let what the master possessed be lost—this cannot be broken; it is my persistence (C8). One must dare to try different styles (C4). I always wanted to create a work for our Yang family (C7).
	Repeated trial and error	Trial and error, starting over	It was more about my own continuous process of trial and error until I was satisfied (C6). I have countless failures; through constant failure, I refine myself, identify the reasons, overcome difficulties, and start again (C3).
	Management and coordination	Communication, coordination, arrangement	After communication, the other party agreed to change the suit to a solid color. It would have been a real problem if we had not communicated and had just woven it as originally planned (C2). Being able to coordinate and arrange production processes, consider the techniques, and even understand costs and consumption—it’s a comprehensive capability. Without this comprehensive ability, it would be difficult to move things forward (C8).

#### 3.3.2 Axial coding across phases

This process involved the continual comparison of various labels, concepts, and categories derived from open coding to uncover underlying relationships among them. Ultimately, these categories were aggregated into main categories. From the 25 subcategories identified through open coding, nine categories were constructed to describe the key sources of self-efficacy for traditional master artisans across the three developmental phases. The connotations of these main categories and the coding process are detailed in Table 4. Based on the categories and their interrelationships, a three-phase dynamic model of the development of self-efficacy among traditional handicraft artisans was ultimately integrated.

**Table 4:** Results of axial coding across the three phases.

Phase	Main category	Subcategory	Connotation
Mastering fundamentals	Affective states	Driven by interest	Spontaneous engagement stemming from pure intrinsic passion and curiosity.
		Familial and national duty	Encompasses supporting a family, fulfilling organizational assignments, maintaining household livelihood, and securing related interests.
		Educational discipline	Instruction by example and strict discipline from elders.
	Vicarious experience	Step-by-step demonstration	Transmission of experience through on-site or documented step-by-step procedures.
		Guidance from others	A timely reminder, demonstration, or suggestion from others at a critical moment.
		Environmental immersion	Being situated within relevant workshops, communities, and daily practices.
	Mastery experience	Past experience	Familiarity with similar processes, identical materials, or related tools.
		Sustained practice	Repeated practice over long periods with high frequency.
Proficiency and application	External support	Recognition through competition	Validating skill level and gaining prestige through competitions, public exhibitions, and bidding evaluations.
		Encouragement and recognition	Recommendations from organizations, acknowledgment from masters, popularity among consumers, and encouragement from family.
		Policy emphasis	Demonstrating importance through organizing relevant activities and formulating related policies.
		Material support	Financial rewards from organizations or material support from family.
	Vicarious experience	Imitation and comparison	Deconstructing and analyzing exemplary works by peers, cultural relics, or client samples to achieve self-breakthrough by discerning differences.
		Seeking help from others	Proactively seeking solutions from peers or masters through offline or online means.
	Affective states	Personal standards	Internalizing standards as intrinsic principles and mental self-discipline.
		External supervision	Establishing explicit standards for constraint and correction through rigorous inspection.
Breakthrough and innovation	Self-regulation	Management and coordination	Operational capabilities involving apprentice task division, material supply, client demands, and benefit management.
		Resource investment	Investment and consumption of one’s own tangible and intangible resources.
		Broad learning and understanding	Proactively absorbing broader knowledge systems, including underlying culture, principles, and aesthetics.
		Technological empowerment	Keeping pace with technological advancements and integrating them into creative work.
		Careful observation	Contemplating the forms and textures of all things, drawing inspiration from natural creation and life details as sources for creative breakthroughs.
	Physiological and affective states	Obsession and addiction	A mental state of being fully immersed and engrossed.
		Courage and belief	Encompasses the boldness to challenge conventions and forge new paths, tenacious conviction, and diligent temperament.
		Desire for recognition	A profound yearning for glory, appreciation, and reputation.
		Physiological states	The quality of the work benefits from visual acuity and manual steadiness.
	Mastery experience	Repeated trial and error	Identifying feasible solutions through exploration amidst repeated setbacks.

### 3.4 Reliability and saturation testing

To ensure the reliability of the analysis, an external auditor familiar with qualitative research but not specialized in self-efficacy theory was invited. Their “theoretical unfamiliarity” provided a fresh perspective, free from the influence of any predetermined analytical framework. First, three transcripts were randomly selected from all interview transcripts using a random number table and provided to the auditor, who had no prior exposure to the research questions or preliminary analytical frameworks. The auditor was then given a clear, open-ended task: (1) thoroughly read the texts; (2) identify all segments related to key events, individuals, internal reflections, or external factors that influenced the artisans’ skill development or changes in confidence, with the validity of such segments contingent upon the same interviewee expressing similar meanings at least twice; (3) assign a concise thematic label to each segment; (4) group similar labels into several core themes. Subsequently, the independent list of themes generated by the auditor was compared item-by-item with the coding framework of this study, and a quantitative assessment was conducted by calculating the proportion of themes that could be mapped onto existing categories. The results showed that all themes identified by the external auditor were already covered by the theoretical model of this study, with no new or disruptive explanatory categories emerging. Minor discussions were held only regarding the classification of a few themes, leading to consensus. This outcome provides support for the reliability of the initial analysis in capturing the core essence of the data.

After the preliminary theoretical model was formed, a stringent saturation test was conducted to examine whether it comprehensively reflected the information within the data. Specifically, after constructing the theoretical model, new participants were added, and additional raw interview data were collected. Subsequently, the research team initiated a complete grounded theory coding procedure anew on this new sample and compared the results with the already constructed core categories, properties, and relationships. The analysis revealed that no new, substantive categories emerged, nor were any previously uncaptured relationships among the existing categories uncovered. This indicates that the phenomena presented by the additional data could all be effectively explained and integrated by the existing theoretical model; therefore, theoretical saturation was achieved.

## 4 Results and discussion

**Figure 1:**
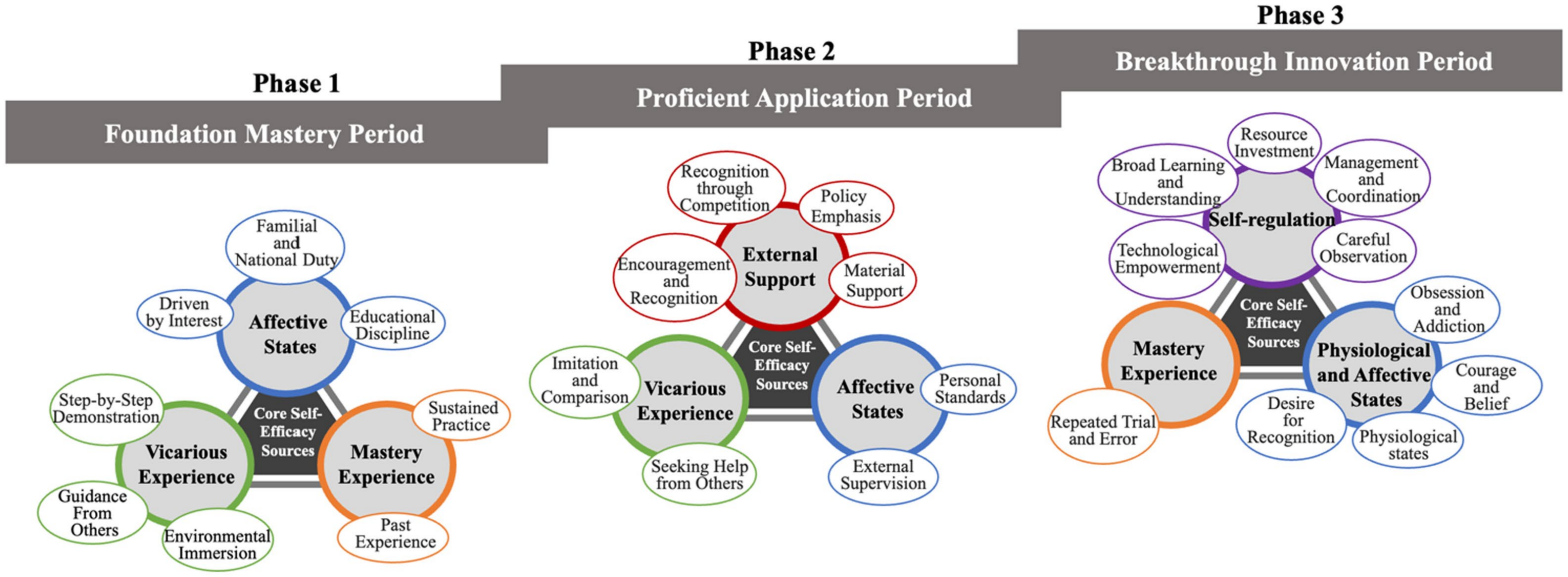
The three-phase developmental model of self-efficacy in traditional handicraft artisans.

Based on the analysis of participant responses, a dynamic model depicting the development of self-efficacy skills among traditional handicraft artisans from apprentice to master was constructed. This model comprises three typical phases and nine key themes, as illustrated in Figure 1. Within each phase, the sources of self-efficacy exhibit distinct differences, reflecting a dynamic developmental trajectory from initiation to proficiency and finally to innovation.

### 4.1 Affective states, vicarious experience, and mastery experience as key sources of self-efficacy in the foundational phase

In the stage where traditional artisans acquire basic skills, the establishment of self-efficacy is not the result of passively receiving external experiences but rather an internal process through which learners actively comprehend, absorb, and transform these elementary experiences. Emotional states, vicarious experiences, and mastery experiences must undergo proactive cognitive processing and meaning construction by the individual to collectively support the psychological transition from observation and imitation to preliminary mastery.

When apprentices learn through vicarious experiences, the emergence of their self-efficacy is closely related to how they comprehend and absorb the observed demonstrations. For step-by-step demonstrations, environmental immersion, and guidance from others to take effect, it is essential for apprentices to internally affirm that these demonstrations are clear, replicable, and relevant to their own situations. For instance, as reflected in the statement of experienced artisan C7, “I would recall how my neighbor did it back then,” confidence stems from actively remembering and drawing upon observations of neighbors during early years in subsequent practice. This implies an internal judgment that the neighbor’s methods can be understood and applied by oneself. Similarly, after receiving suggestions from teachers or photographers (C7, C9), the encouraging effect arises from their interpretation of such external guidance as “effective and crucial” assistance. If an individual perceives the demonstrated content as overly advanced or irrelevant, vicarious experiences may struggle to translate into tangible confidence. Thus, the significance of vicarious experiences consistently relies on the individual’s internal validation of their learnability and practical applicability.

Affective states constitute the internal support for sustained learning, encompassing personal interest, familial or communal responsibility, and educational discipline. Bandura’s concept of “physiological states” was not mentioned by the artisans at this stage and thus appears less salient. The passion for the craft itself enables artisans to endure the initial monotony because it is cognitively transformed into an intrinsic sense of pleasure and reward, reframing repetitive practice as “exploration” and “dedication.” For instance, as C1 and C8, respectively, noted, “Having this inner fondness makes it feel less difficult.” A more resilient form of support often stems from the internalization and identification with external responsibilities such as familial heritage, assignments from trade organizations, or the duty to provide for one’s family. These external requirements are not in themselves emotions, but the craftsmen transform them into a series of deep-seated emotions by imbuing them with personal meaning. As experienced artisan C5 expressed, “I can practice craftsmanship while taking care of my family. That was the reason at the time; otherwise, I wouldn’t have chosen this.” It can be seen that C5 did not view “earning a living” and “caring for the family” as two conflicting tasks; instead, he perceived the possibility of achieving both through the craft of handicrafts. It is precisely this cognitive confirmation of “having it both ways” that transforms the external requirement of family responsibility into an internal sense of relief and certainty. He binds this craft to the family role of “becoming a father/husband who can support the family and be present,” thereby endowing his career choice with personal meaning, which in turn fosters a sense of value and belonging. In daily practice, this emotional confirmation, transformed from a sense of responsibility, becomes a stable internal drive. Most artisans in Gujarat, for example, transform their inherent skills into professional capabilities to increase household income (Shah and Patel, 2017). Meanwhile, experienced artisan C3 mentioned, “My mother cultivated in me the habit of being independent and self-reliant, so when I encounter difficulties, I never think of escaping. This applies to everything.” This statement reveals how educational discipline is transformed into enduring emotional states through cognitive appraisal. First, C3 interprets his mother’s past “cultivation” as a form of well-intentioned shaping, rather than coercion or control. This positive reappraisal of the source of discipline generates in him, during recall, a sense of identification with and gratitude towards his mother. More importantly, by internalizing “independence and self-reliance” from his mother’s requirement into “my habit,” it signifies that this discipline has been integrated into his self-identity. Consequently, when facing difficulties, what drives him to “never think of escaping” is no longer passive compliance with his mother’s teachings, but a profound sense of self-affirmation: “I am a person shaped this way, and I should be this kind of person.” This sense of self-esteem and the need for self-consistency, derived from the internalization of discipline, makes perseverance an emotional necessity. Escape would imply not just a behavioral retreat, but a betrayal of his self-identity. It is precisely this internalized emotional force that enables C3 to maintain consistent resilience in the face of any difficulty. This internal process, of interpreting external expectations or pressures as personal meaning, feasible pathways, or identity traits, lies at the heart of how emotional states are transformed into steadfast perseverance.

Mastery experience, accumulated through repeated personal practice, manifests as learners gradually gain a sense of control and successful experience with the craft through prolonged, frequent practice and exploration of similar procedures, identical materials, and related tools. This process solidifies a stable belief that “I can learn this.”

### 4.2 External support, vicarious experience, and affective states as key sources of self-efficacy in the proficiency phase

As traditional craftspeople advance into the proficiency phase of skilled application, the consolidation and enhancement of their self-efficacy rely mainly on external support, vicarious experience, and affective states. These three key sources jointly underpin the formation of a robust and enduring professional confidence during this phase.

External support in this phase manifests through four key subcategories: recognition through competitions and exhibitions, encouragement and affirmation, policy prioritization, and material assistance. These elements serve as vital reinforcements for artisans’ beliefs in their own capabilities. On one hand, the recognition and reputation gained from public activities such as competitions, exhibitions, awards, and bidding processes strengthen artisans’ confidence because they interpret these achievements as objective validation of their professional competence. This understanding transforms into an inner conviction that their capabilities have been recognized and affirmed. For instance, artisan C7 described, “Exhibitions came one after another, and my works were published, so my state of mind was different at that time, and I grew increasingly motivated.” This statement illustrates how external opportunities for showcasing work are internalized as sources of personal accomplishment and sustained drive. On the other hand, the recognition and recommendations from industry organizations, continuous acknowledgment from masters, positive feedback from consumers, and emotional encouragement from family also need to be internalized by the individual as affirmation of their efforts and direction, thus forming stable psychological support. As demonstrated by artisan C1, who, upon receiving recognition from his master for his dedication to learning, perceived it as positive affirmation of his attitude. This network helps them maintain confidence in their ability to overcome complex challenges. Tangible policy support and material assistance from institutions or families further translate into practical conditions that allow for sustained investment and focused creation. It is noteworthy that the classic source of “social persuasion” in Bandura’s theory manifests within the context of this field in the form of external support. This external support is not limited to oral encouragement but also takes the form of substantive material support from groups such as families, government entities, and clients.

Vicarious learning in this phase becomes more proactive and in-depth, specifically involving the subcategories of comparative imitation and seeking help. Artisans absorb the essence of others’ work by analyzing masterpieces by peers, historical artifacts, or exemplary samples provided by clients, breaking through their own limitations through meticulous comparison. This process relies on their proactive construction of connections between others’ experiences and their own needs. For instance, when presented with reference materials provided by a client, artisan C8 remarked, “Having a reference makes it somewhat manageable.” This reflects an understanding of the reference’s executability, grounded in his own experience. Conversely, abstract and ambiguous requirements were perceived as difficult to grasp. Notably, this process often benefits from the analytical assistance of researchers or relevant experts. For instance, a senior artisan (C1) mentioned purchasing and studying monographs published by museum researchers, which provide magnified microscopic analyses of specific categories of historical masterpieces. When encountering technical impasses, they proactively seek solutions through industry exchanges or master-apprentice channels, ultimately transforming others’ successful experiences into cognitive frameworks for addressing new problems.

Affective states in this phase deepen into the subcategories of self-imposed standards and external scrutiny. Furthermore, Bandura’s concept of “physiological states” was not mentioned by the artisans at this stage and thus appears less salient. On the one hand, the high standards of the industry, the rigorous demands of masters, and the inspection systems within workshops are accepted and internalized by individuals as part of their professional attitude, fostering a habitual mindset of “doing one’s work conscientiously and solidly.” On the other hand, these external norms are further integrated with personal artistic pursuits, evolving into an internal drive of “perfectionism.” As artisan C8 noted, “The master thought it was generally acceptable, but I always felt it could be executed more precisely.” This meticulousness is no longer driven by external pressure but stems from an inner self-defined and persistent pursuit of “precision” and “perfection”—that is, “what matters more is the standard within oneself.” In the field of traditional craftsmanship, a perfectionist mindset can positively influence the maker (Bhat and Mathews, 2024). This process of merging external oversight with internal standards transforms emotional states from passive responses into active, sustained self-motivation, thereby reinforcing a deep-seated belief in the mastery of the craft.

### 4.3 Self-regulation, physiological and affective states, and mastery experience as key sources of self-efficacy in the innovative breakthrough phase

When traditional craftspeople evolve into the innovative breakthrough phase, the key sources of their self-efficacy are primarily reflected in the interaction between self-regulation, Physiological and affective states, and mastery experience.

Self-regulation is the core characteristic of this stage. Its significance for efficacy does not stem from the mere possession of resources but rather from how artisans understand and utilize this autonomy to define the relationship between self and creation. It encompasses five dimensions: management and coordination, resource investment, extensive learning and comprehension, technological empowerment, and meticulous observation. Recent scholarly discussions have dialectically examined the disempowering and empowering impacts involved in co-design processes with artisans, covering emotional, cognitive, behavioral, and relational dimensions (Wang et al., 2023). Zimmerman (2000) pointed out that self-efficacy beliefs interact with individuals’ self-regulated learning processes, such that self-efficacy influences learners’ choice of and persistence in learning strategies, while effective self-regulatory practices in turn reinforce efficacy beliefs. Wang and Ma (2024) posit that by positioning craftspeople equipped with modern design knowledge as the central driving force and leveraging their internal knowledge integration and transformation as the core dynamic, multi-agent collaboration can effectively mitigate the risk of marginalizing artisans during design intervention processes. The core of this proposal lies in establishing and reinforcing the agency and decision-making authority of handicraft artisans in innovative design, thereby fundamentally ensuring their central role in practical collaboration, rather than a passively executing one. Building upon this research context, this study further proposes that self-regulation constitutes a core characteristic of self-efficacy sources for artisans during the breakthrough and innovation phase. The essence of this characteristic lies in the artisans’ cognitive shift from an “executor” to a “director” of their work, which they internalize as a fundamental creative conviction. For instance, artisan C7’s statement, “I wanted to take charge and do what I desired, so I self-funded this creative project,” clearly demonstrates that autonomy has evolved from a mere aspiration into a firm stance characterized by the willingness to bear costs and act accordingly. This sense of control is reflected in their ability to coordinate complex processes, encompassing the management of tasks ranging from apprentice allocation and material distribution to client communication and operational profitability, as well as the strategic investment of tangible and intangible resources such as time, energy, and capital. Furthermore, this characteristic emerges as a novel efficacy source within this phase, emphasizing autonomous authority over the creative process. It encompasses five distinct subcategories: management and coordination, resource investment, broad learning and understanding, technological empowerment, and careful observation. Artisans are able to coordinate and manage the entire workflow, from apprentice task allocation and material procurement to client communication and operational efficiency. They strategically invest tangible and intangible personal resources, including time, energy, finances, and even health. Actively constructing cross-disciplinary knowledge networks, they deepen their understanding of the cultural context, scientific principles, and aesthetic systems underlying their craft. They consciously integrate contemporary technological methods into traditional practices to achieve creative extension and transformation. Concurrently, they continually draw inspiration from natural forms, material textures, and details of daily life, transforming observation itself into a mode of creative thinking, thereby continually expanding the boundaries of their work. This high degree of cognitive autonomy and decision-making authority enables individuals to consciously plan creative directions, integrate and allocate resources, and maintain a sense of control over both process and outcome even amidst uncertainty. It thereby becomes an exceptionally significant source of self-efficacy during the breakthrough and innovation phase. Its fundamental distinction from mastery experience lies in their respective focal points. Mastery experience emphasizes confidence in skills developed through past repetitive practice, representing assurance at the level of capability. In contrast, self-regulation focuses on the authority and agency to command the creative process and environmental resources, constituting a belief at the level of authority and proactive agency. During the breakthrough and innovation phase, artisans require not only technical proficiency but, more critically, the capacity for self-regulation to explore new domains and experiment with novel methods, thereby accomplishing the transition from production to creation. Consequently, it is of paramount importance within this advanced phase.

On the level of physiological and affective states, the driving forces in this phase often manifest as obsession/addiction, courage and conviction, a desire for recognition, and physiological states. Craftspeople frequently enter a state of total immersion and enjoyment; this near-addictive engagement enables them to persistently face lengthy and complex creative processes. Senior artisan C7 noted that he even thinks about the craft while sleeping. This near-obsessive engagement is interpreted by artisans as a natural state in which creative inspiration profoundly intertwines with their personal life rhythms. It is even imbued with positive meaning, such as being seen as “divine inspiration,” thereby romanticizing arduous contemplation as an integral part of the creative process. Deeper convictions are often linked to personal identity and a sense of history, such as “reverence for the culture… is my obsession,” or “I have always wanted to create a work for our Yang family.” These emotions transcend ordinary responsibility, evolving into an inner pillar that imbues creation with historical significance and familial purpose. Furthermore, they possess the courage to challenge conventions and forge their own paths. Their convictions often carry a quality of steadfast dedication, complemented by diligent and unwavering practical will. Artisans in this phase harbor a profound desire for industry recognition, social appreciation, and historical legacy. This aspiration to leave a mark further strengthens their resilience in persisting through difficulties during exploration. At the same time, artisans develop a more profound awareness of their physical condition, which significantly influences their efficacy judgments. Statements such as “I was young then, with good eyesight—that’s why I could complete such a work” and comparisons with their current manual abilities not only describe objective circumstances but also imply a deep recognition and acceptance of the relationship between “physical capital” and the “peak creative period.” This understanding leads them to adapt their creative methods or transform nostalgia for past physical capabilities into affirmation and appreciation of the achievements made during that specific period, rather than mere regret.

Although self-regulation, physiological and affective drive play significant roles, mastery experience remains irreplaceable in this phase. Mastery experience primarily manifests as repeated trial and error and path exploration in uncharted territory. Its efficacy value derives from how artisans interpret failure and success. Trial and error alone does not automatically build confidence; the key lies in the cognitive processing through which artisans actively “extract lessons from themselves and identify causes” from the process of “encountering numerous failures,” thereby reframing failure as a necessary learning step toward success. The statement, “It is more about continuously engaging in trial and error until I achieve what satisfies me,” reveals the essence of mastery experience at this stage: confidence does not stem from flawless success but from the complete, self-directed learning cycle of “achieving self-set standards through persistent, self-guided trial and error.” Each successful foray into the unknown deepens their inner conviction in their ability to solve new problems and achieve innovation.

## 5 Conclusions and implications

Drawing upon the phased patterns of self-efficacy sources elucidated by the aforementioned model, this study proposes program design strategies for cultivating complex practical competencies. The core principle should be the dynamic alignment with the psychological developmental needs of traditional handicraft artisans throughout their learning trajectory.

During the foundational mastery phase, the central challenge for artisans is overcoming initial anxiety and establishing fundamental confidence in their learning capacity. Given that their self-efficacy primarily derives from positive emotional states, vicarious experience, and initial mastery experiences, external support should focus on reducing cognitive load and emotional barriers. Consequently, the key to program design lies not in providing entirely open exploration space, but in constructing a structured and predictable learning environment. By offering clear step-by-step demonstrations, modular micro-tasks, and immediate positive feedback, learners can attain predictable successful experiences. This facilitates the rapid accumulation of foundational mastery experiences, thereby solidifying the cornerstone of confidence at the entry level.

When artisans progress to the phase requiring flexible skill application in complex contexts, the maintenance and enhancement of their efficacy depend more substantially on external support systems, in-depth vicarious experiences, and emotional regulation within professional settings. This indicates that technical practice alone is insufficient; learning must be embedded within authentic or simulated social practice. Therefore, the focus of educational intervention should shift toward building a supportive platform that connects mentors, peers, clients, and broader industry resources. Through the introduction of real-world projects, provision of industry feedback, and creation of opportunities for exchange and comparison, artisans can validate and consolidate their capabilities via external recognition, collaboration, and challenges. This process completes the transformation of confidence from knowing how to execute tasks to being capable of effective application.

For learners reaching the innovation phase, the core psychological task is achieving an identity shift from technical executor to conceptual creator. This study finds that the central efficacy source at this stage is self-regulation over the creative process. This transcends confidence based on proficient skill, representing a higher-order belief concerning creative autonomy and cultural agency, operating alongside sustained positive psychological states and mastery experiences. Accordingly, external support must transition from providing resources to restoring agency and co-constructing meaning. Ideal educational design should aim to build a professional creative community led by the artisans themselves. By granting them project decision-making authority, resource allocation power, and providing deep dialogue and endorsement at academic and cultural levels for their innovative explorations, such a community supports the translation of internal creative impulses into socially and culturally valuable outcomes.

In summary, the model constructed in this study not only provides a detailed map for understanding the psychological pathway of traditional handicraft transmission but also offers cross-disciplinary insights through its core principle of aligning psychological dynamics with developmental phases. It indicates a critical direction for contemporary STEM education and various practical training programs that emphasize innovation cultivation. Fostering exceptional talent requires not only planning the progressive advancement of knowledge and skills but also meticulously designing a psychological support system that evolves in synchrony, thereby supporting the synergistic development of cognition, emotion, and behavior.

Future research could apply this model as an analytical framework to specific STEM program designs and empirically test its effectiveness. However, this study has several limitations. First, although theoretical saturation was achieved through interviews with 10 participants, the sample cannot encompass the full diversity and complexity of traditional handicraft categories. Therefore, expanding the sample size is recommended. Furthermore, due to the open-ended nature of the responses, which required senior artisans to recall the reasons for their efficacy judgments without guidance from the researcher, measuring sources solely through retrospective recollection offers a limited perspective on the information artisans actually used when judging their self-efficacy. This is because individuals may underestimate critical factors or overestimate factors that had little to no actual impact when recalling influences on their self-efficacy beliefs (Usher and Pajares, 2008). Furthermore, these accounts may also be influenced by memory reconstruction and participants’ current status as senior and successful practitioners. Therefore, it is recommended to employ a combined approach of participatory observation and unstructured interviews. Through prolonged understanding and communication, a more comprehensive exploration of these self-efficacy sources can be achieved. Allowing interviewees to rank the importance of identified sources themselves could further aid in determining key information. Finally, it should be noted that, in presenting the original statement examples, we have aimed to depict the specific actions, contextual environments, psychological states, and other related aspects of the respondents when facing difficulties. However, as the original utterances underwent a translation process from dialect to Mandarin and then to English, many subtle modal particles and affective cues were lost in the process. Additionally, due to constraints on text length, many paralinguistic and gestural nuances could not be fully captured. Therefore, the representation of the respondents’ cognitive dimensions remains relatively limited.

## Data Availability

The datasets presented in this article are not readily available because the original data supporting the findings of this study are not publicly available in order to protect the privacy of the research participants and to comply with the confidentiality agreement approved by the relevant ethics committee at Jiangnan University. Requests to access the datasets should be directed to 7220306003@stu.jiangnan.edu.cn.
